# Hepatic arterialization can predict the development of collateral veins in patients with HCV-related liver disease

**DOI:** 10.1007/s40477-018-0323-4

**Published:** 2018-10-05

**Authors:** Noritaka Wakui, Hidenari Nagai, Yu Ogino, Kojiro Kobayashi, Daigo Matsui, Takanori Mukozu, Yasushi Matsukiyo, Teppei Matsui, Yasuko Daido, Koichi Momiyama, Mie Shinohara, Takahide Kudo, Kenichi Maruyama, Yasukiyo Sumino, Yoshinori Igarashi

**Affiliations:** 1 Division of Gastroenterology and Hepatology, Department of Internal Medicine (Omori), School of Medicine, Faculty of Medicine, Toho University, 6-11-1 Omori-nishi, Ota-ku, Tokyo, 143-8541 Japan; 2 Division of Clinical Functional Physiology, Toho University Omori Medical Center, 6-11-1 Omori-nishi, Ota-ku, Tokyo, 143-8541 Japan; 3 Department of Gastroenterology and Hepatology, Japan Community Health Care Organization (JCHO) Tokyo Kamata Hospital, 2-19-2 Minami-kamata, Ota-ku, Tokyo, 144-0035 Japan

**Keywords:** Arrival time parametric imaging, Contrast-enhanced ultrasonography, Hepatitis C, Collateral vein, Esophageal varices, Arterialization

## Abstract

**Purpose:**

Arrival time parametric imaging (At-PI) using contrast-enhanced ultrasonography (CEUS) is a procedure for evaluating liver disease progression in chronic hepatitis C infection (CHC). We investigated At-PI diagnostic efficacy in predicting development of collateral veins.

**Methods:**

In total, 171 CHC patients underwent CEUS and upper gastrointestinal (UGI) endoscopy before liver biopsy. Conventional US was performed before CEUS to identify paraumbilical veins (PV) or splenorenal shunts (SRS). After intravenous perflubutane, contrast dynamics of liver segments 5–6 and the right kidney were saved as raw data. At-PI image ratio of red (ROR) pixels to the entire liver was analyzed. Receiver operating characteristic (ROC) curves were generated to investigate the utility of At-PI for collateral vein identification.

**Results:**

Conventional US revealed PV in two patients and SRS in five patients; UGI endoscopy detected esophageal varices (EV) in eight patients. Diagnostic capability of At-PI for detecting PV, SRS, and EV was satisfactory, and high for PV and SRS [PV; area under the ROC curve (AUROC) 0.929, cutoff value 77.9%, SRS; AUROC 0.970, cutoff value 82.0%, EV; AUROC 0.883, cutoff value 66.9%].

**Conclusions:**

Evaluation of hepatic arterialization by At-PI was useful for predicting collateral vein development in CHC patients.

## Sommario

**Scopo:**

L’imaging parametrico con calcolo del tempo di arrivo (At-PI) mediante l’uso dell’ecografia con mezzo di contrasto ( CEUS) è una procedura che può valutare la progressione della malattia cronica di fegato in corso di epatite HCV relata (CHC). Gli Autori hanno indagato l’efficacia diagnostica di At-PI nel predire lo sviluppo di circoli collaterali.

**Metodi:**

In totale 171 pazienti sono stati sottoposti a CEUS e ad endoscopia del tratto digestivo superiore (UGI) prima della biopsia epatica. Un’ecografia convenzionale è stata eseguita prima della CEUS per identificare pervietà della v. ombelicale (PV) o di shunt spleno-renale spontaneo (SRS). Dopo iniezione e.v. di perflubutano sono stati salvati i dati grezzi della dinamica del m.d.c. raccolti dai segmenti 5 e 6. E’ stato inoltre analizzato il rapporto dell’immagine At-pi con i pixel rossi (ROR) dell’intero fegato. Sono state ottenute delle curve ROC per valutare l’utilità della At-PI nell’identificare circoli collaterali.

**Risultati:**

L’ecografia di base ha identificato PV in due pazienti e SRS in cinque pazienti; UGI ha diagnosticato varici esofagee in otto pazienti. La capacità diagnostica di At-PI per identificare PV, SRS e varici esofagee è stata soddisfacente ed elevata per PV ed SRS (PV : area sotto la curva ROC (AUROC) = 0,929 con valore di cut-off = 77,9%; SRS : AUROC = 0,970 con cut-off = 82;0% ; varici esofagee : AUROC = 0,883 con cut-off = 66,9 ).

**Conclusioni:**

La valutazione dell’arterializzazione epatica mediante At-PI è stata utile nel predire circoli collaterali spontanei nei pazienti con malattia cronica di fegato HCV relata.

## Introduction

The liver receives dual blood supply from the hepatic portal vein and the hepatic artery. The portal vein provides approximately 70–80% of the supply, carrying nutrients and various other substances to the liver. The remaining 20–30% of the blood supply comes via the hepatic artery and mainly supplies the biliary system. When the liver is infected with hepatitis C virus, the pathology progresses from hepatitis to cirrhosis. Due to the intrinsic low-pressure system, the hepatic portal vein is more susceptible to changes in liver tissue, compared with the hepatic artery.

Consequently, the balance of blood flow between the hepatic artery and portal vein shifts from portal venous dominant to hepatic arterial dominant as liver disease progresses [1–4], causing complications such as portal hypertension (PH). If diagnostic imaging can be used to visualize and quantify the ever-changing hepatic blood flow during disease progression, it will be possible to monitor the development and onset of liver disease noninvasively, including the complication of PH [5].

We hypothesize that it is best to use contrasts of liver and kidneys, which are supplied only by the arterial system, to capture changes specific to hepatic hemodynamics. Wakui et al. investigated the progression of liver disease, who developed a technique called arrival time parametric imaging (At-PI) for contrasting of the liver and kidney to determine the arrival time of the contrast agent by superimposing a temporal color map on a B-mode image.

Wakui et al. showed that At-PI enables diagnosis of progression of liver fibrosis [6], and PH is known to worsen as liver fibrosis progresses [7]. PH is a frequent complication of cirrhosis, contributing to the development of paraumbilical veins (PV), esophageal varices (EV), and splenorenal shunts (SRS). The best available method for the assessment of PH is measurement of the hepatic venous pressure gradient (HVPG). However, HVPG measurement is limited to highly specialized centers and requires extensive experience, limiting its routine use [8].

Therefore, in this study, we investigated whether AT-PI, a noninvasive diagnostic modality, predicts the development of collateral veins in response to progression of liver fibrosis.

## Patients and methods

### Patients

We recruited 179 patients with CHC infection between June 2007 and June 2015. The diagnosis of CHC was made based on positive hepatitis C virus (HCV)-RNA quantification by TaqManPCR (Invitrogen, Carlsbad, CA), and the absence of hepatitis B surface antigen and hepatitis B core antibody. Patients with a daily alcohol consumption of > 80 g, heart or kidney disease, hepatic tumor, or portal vein thrombosis were excluded from the study. Patients with unclear imaging results due to, for example, narrow intercostal spaces, were also excluded.

After excluding 8 patients (narrow intercostal spaces, *n* = 5; inability to hold breath, *n* = 3), 171 patients comprising 99 men and 72 women aged 56 ± 14 (range 21–85) years were included in the analysis. The study protocol was in accordance with the Declaration of Helsinki 1975, as revised in 2008, and was approved by the ethics committee of our institution (No. 26–227). Written informed consent was obtained from all participants.

### Conventional US for the verification of collateral veins

US was performed using a Canon AplioXG (SSA-790A; Canon Medical Systems, Tochigi, Japan) with a 3.75-MHz convex array probe (PVT-375BT; Toshiba Medical Systems). All patients fasted overnight before the examination. B-mode imaging was performed to verify the presence of PV and SRS.

### Contrast-enhanced ultrasonography

CEUS was performed from the right intercostal space using the same US device and probe used in conventional US. The mechanical index and frame rate were set to 0.22–0.29 and 15–18 frames/s, respectively. Images showing liver parenchyma of the right hepatic lobe (segment 5 or 6) and the right kidney were used in the analysis. Focus was set to 6–8 cm to cover the whole kidney. Participants were examined in the supine position with the right arm elevated above the head and instructed to hold their breath. All patients fasted overnight before the examination. After setting imaging parameters, the recommended dose (0.015 mL/kg) of the second-generation CEUS agent perflubutane [9] (Sonazoid; GE Healthcare, Oslo, Norway) was administered as a bolus via the median cubital vein at a rate of 1 mL/s and flushed with 10 mL of normal saline. Cine acquisition was started at the beginning of saline flush. Data generated for the first 40 s were saved as raw data in the system hardware. All US examinations were performed by an independent examiner with over 24 years of experience as an ultrasonographer and who was blinded to patient characteristics.

### Arrival time parametric imaging

The software interfaced with the ultrasound system was used to generate At-PI images from stored video clips. By simply selecting the renal parenchyma as the region of interest, the system set the point at which 80% of the region of interest was contrasted as time 0 and sequentially calculated the arrival time in individual pixels of the hepatic parenchyma. The system then automatically created and superimposed a color map on a B-mode image. The difference in the arrival times of arterial and subsequent portal venous blood to the liver was reported to be 5 s [10]. From the freely selectable display colors, we therefore used red and yellow to display pixels arriving at 0 to < 5 s and at ≥ 5 to 10 s, respectively. In other words, red and yellow indicate the liver parenchyma supplied by blood through the arterial and venous routes, respectively.

### Measurement of contrasted areas

To quantitatively evaluate the obtained At-PI data, the ratio of the area of red pixels with shorter arrival times to the area of pixels in the entire contrast-enhanced area was calculated as the ratio of red (ROR), using ImageJ version 1.42 image analysis software (Wayne Rasband, National Institutes of Health, Bethesda, MD).

A higher ROR means that the arrival time of the contrast agent in the liver is closer to that in the kidney; in other words, a wider area of the liver parenchyma received the contrast agent from the arterial route, indicating a shift in the arterial portal blood flow balance toward arterial dominance in the liver. Two physicians calculated the ROR. Both were physicians trained in the use and interpretation of contrast agents in the liver.

To calculate the ROR, ImageJ was used to select and measure only the red areas in the liver parenchyma on arrival time parametric images. Next, the entire area of contrast enhancement in the liver parenchyma was displayed in the same color to measure the area. Lastly, the calculation of ROR was performed by two physicians. Both were physicians trained in the use and interpretation of contrast agents in the liver. They were not involved in sonographic scanning and were blinded to the identification, clinical history, and other imaging findings of the patients. Both physicians jointly analyzed the ROR. Using ImageJ software, one physician measured the ratio, and the other examined each case and evaluated the accuracy of the ratio measurement performed by the other physician.

### Upper gastrointestinal endoscopy

Upper gastrointestinal endoscopy was performed using the GIF-Q260 system (Olympus Optical, Ltd., Tokyo, Japan). Endoscopic findings of EV were classified according to the General Rules for Recording Endoscopic Findings set by the Japan Research Society for Portal Hypertension [11]: form 0 (non); form 1 (straight), form 2 (winding), and form 3 (nodule-beaded), corresponding to the grades of small, medium, and large, respectively. Forms 1–3 were considered positive for EV.

### Diagnostic capability of the ratio of red for liver fibrosis and collateral veins (esophageal varices, paraumbilical veins, splenorenal shunts)

Liver biopsies were performed after sonography with a 16-gauge liver biopsy needle (Core II™ semiautomatic biopsy instrument; InterV Clinical Products, Dartmouth, MA). Specimens were obtained from the anterior segment of the right lobe under US guidance and fixed in 10% formalin, embedded in paraffin, sectioned, and stained with hematoxylin–eosin and azan for histologic evaluation. All biopsy specimens were evaluated by a single experienced pathologist blinded to the clinical conditions of the patients. Pathologic liver fibrosis was evaluated using the Metavir scoring system [12]. Fibrosis was staged as follows: F0, no fibrosis; F1, portal fibrosis without septa; F2, portal fibrosis with few septa; F3, numerous septa without cirrhosis; and F4, cirrhosis.

After liver biopsy, the ROR in each patient was compared with F stage (F0–4), EV (form 0 versus forms 1–3), PV, and SRS to elucidate the diagnostic capability of At-PI for liver fibrosis, EV, PV, SRS, and any of the three collateral veins. US imaging and upper GI endoscopy were performed for each patient within 3 months.

### Statistical analysis

The Jonckheere–Terpstra trend test and Steel–Dwass test were used for comparative analysis of F stage and ROR. In addition, receiver operating characteristics curves were used to determine the optimum cutoff value and the area under the receiver operating characteristic (AUROC) curve for the diagnosis of F stage and EV, PV and SRS. Statistical analysis was performed using Excel 2012 (SSRI Co., Tokyo, Japan) statistical analysis software, with significance set at *p* < 0.05.

## Results

### Characteristics of patients

The clinical and biochemical characteristics of the patients (*n* = 171) are summarized in Table 1. The stage of fibrosis was 0–1 in 84 patients, 2 in 45 patients, 3 in 26 patients, and 4 in 16 patients. Aspartate transaminase (IU/L) was 41 ± 30 in F0–1, 60 ± 35 in F2, 85 ± 43 in F3, and 96 ± 53 in F4. Alanine transaminase (IU/L) was 49 ± 45 in F0–1, 67 ± 48 in F2, 88 ± 50 in F3, and 79 ± 44 in F4. Platelet count (× 10^4^/µL) was 19.0 ± 5.8 in F0–1, 16.4 ± 5.4 in F2, 12.0 ± 4.9 in F3, and 8.9 ± 3.2 in F4. Total bilirubin (mg/dL) was 0.7 ± 0.3 in F0–1, 0.8 ± 0.9 in F2, 0.8 ± 0.3 in F3, and 0.9 ± 0.3 in F4. Albumin (g/dL) was 4.2 ± 0.6 in F0–1, 4.0 ± 0.4 in F2, 3.9 ± 0.4 in F3, and 3.4 ± 0.4 in F4. Total cholesterol (mg/dL) was 174.1 ± 42.2 in F0–1, 159.3 ± 28.0 in F2, 159.0 ± 37.8 in F3, and 143.8 ± 24.6 in F4. Prothrombin time (% of normal) was 100.4 ± 22.5 in F0–1, 92.4 ± 12.8 in F2, 86.3 ± 12.4 in F3, and 80.0 ± 7.9 in F4.

**Table 1:** Characteristics of 171 patients with HCV-related liver disease

Fibrosis stage	F0–1	F2	F3	F4
Number	84	45	26	16
Sex (male/female)	51/33	21/24	15/11	12/4
Age (years)	52 ± 13	59 ± 12	60 ± 7	58 ± 12
AST (U/L)	41 ± 30	60 ± 35	85 ± 43	96 ± 53
ALT (U/L)	49 ± 45	67 ± 48	88 ± 50	79 ± 44
Platelet count (× 10^4^/µL)	19.0 ± 5.8	16.4 ± 5.4	12.0 ± 4.9	8.9 ± 3.2
Total bilirubin (mg/dL)	0.7 ± 0.3	0.8 ± 0.9	0.8 ± 0.3	0.9 ± 0.3
Albumin (g/dL)	4.2 ± 0.6	4.0 ± 0.4	3.9 ± 0.4	3.4 ± 0.4
Total cholesterol (mg/dL)	174.1 ± 42.2	159.3 ± 28.0	159.0 ± 37.8	143.8 ± 24.6
Prothrombin time (% of normal)	100.4 ± 22.5	92.4 ± 12.8	86.3 ± 12.4	80.0 ± 7.9

Values are expressed as the mean ± standard deviation

*AST* aspartate transaminase, *ALT* alanine aminotransferase, *NH*_*3*_ ammonia

### Multiple comparisons of ROR from different stages of liver fibrosis

**Fig. 1:**
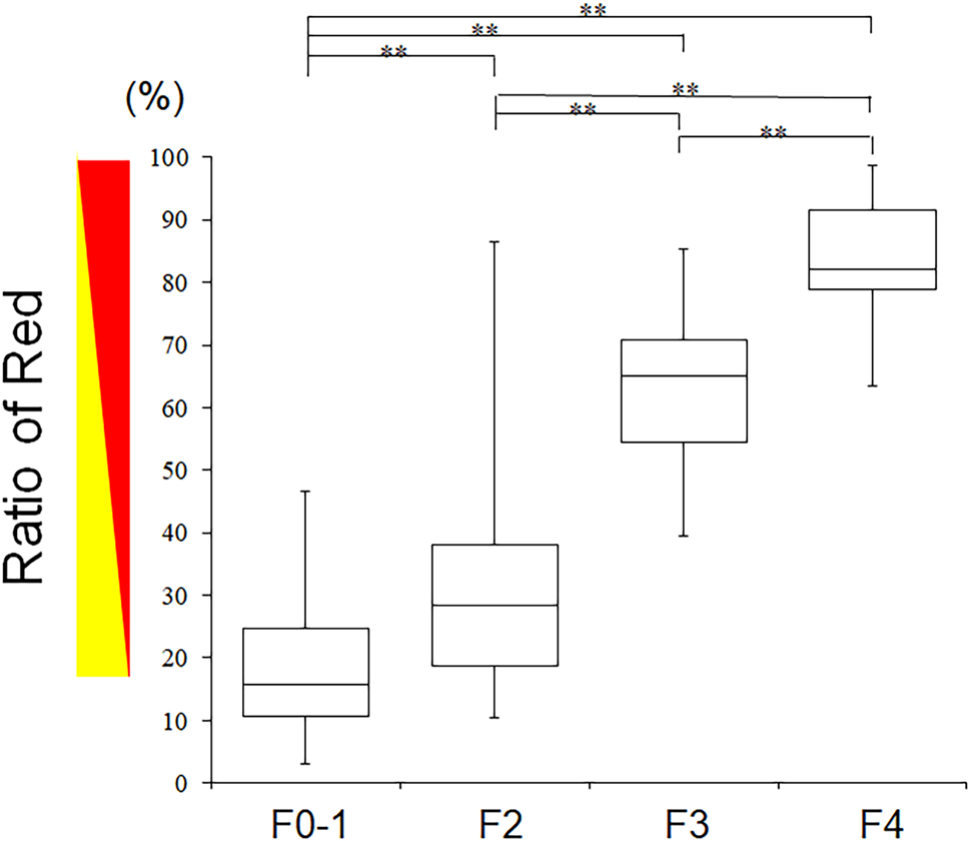
Parameter analysis measured by arrival time parametric imaging for each fibrosis stage. Box plots of each arrival time parametric imaging value corresponding to fibrosis stages F0–4. Top and bottom of each box indicates the first and third quartiles. Length of the box represents the interquartile range within which 50% of values are located. Line through the middle of each box represents the median value. F0–4, *n* = 171 [color online]

The ROR in each F stage was as follows: 18.0 ± 10.3% for F0–1, 31.3 ± 18.2% for F2, 60.9 ± 14.9% for F3, and 81.4 ± 11.8% for F4. The ROR variation trend in F0–4 patients showed a significant, increasing trend in the ROR with increasing F stage (*p* < 0.0001). Multiple comparisons showed significant differences between F0–1 and F2 (*p* < 0.01), F0–1 and F3 (*p* < 0.01), F0–1 and F4 (*p* < 0.01), F2 and F3 (*p* < 0.01), F2 and F4 (*p* < 0.01), F3 and F4 (*p* < 0.01), demonstrating that ROR increased significantly with fibrosis progression (Fig. 1).

### Diagnostic capability for liver fibrosis based on ROC curves

**Fig. 2:**
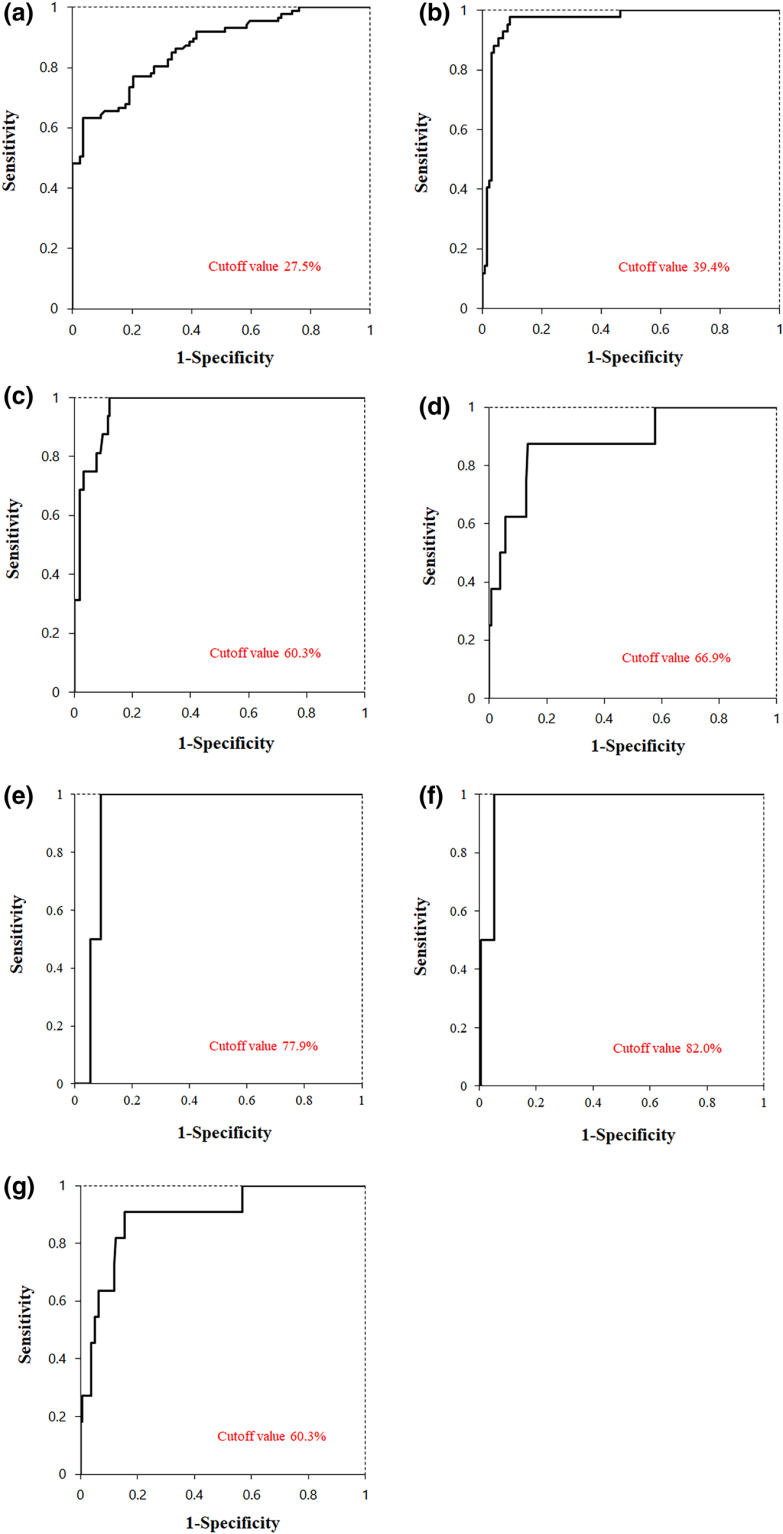
Receiver operating characteristic (ROC) curves. **a** ROC curve for arrival time parametric imaging diagnosis of stage ≥ F2 liver fibrosis [area under curve (AUROC) = 0.867]. **b** ROC curve for arrival time parametric imaging diagnosis of stage ≥ F3 liver fibrosis (AUROC = 0.963). **c** ROC curve for arrival time parametric imaging diagnosis of stage ≥ F4 liver fibrosis (AUROC = 0.965). **d** ROC curve for arrival time parametric imaging diagnosis of EV (≥ form 1) (AUROC = 0.883). **e** ROC curve for arrival time parametric imaging diagnosis of PV (AUROC = 0.929). **f** ROC curve for arrival time parametric imaging diagnosis of SRS (AUROC = 0.970). **g** ROC curve for arrival time parametric imaging diagnosis of collateral veins (AUROC = 0.895)

The cutoff value and AUROC were 27.5 and 0.867 for the diagnosis of fibrosis stages F0–1 and ≥ F2, respectively, and 39.4 and 0.963 for stages F0–2 and ≥ F3, respectively, and 60.3 and 0.965 for stages F0–3 and ≥ F4, respectively. When these values were used to diagnose cases ≥ F2 and ≥ F3 and ≥ F4 stages in At-PI, the sensitivity and specificity were, respectively, 0.770 and 0.797 for cases ≥ F2 and 0.976 and 0.907 for cases ≥ F3 and 1.000 and 0.877 for cases ≥ F4 (Fig. 2a–c).

### Diagnostic capability for esophageal varices based on ROC curves

Eight of the 171 patients (4.7%) were diagnosed with EV (form 1, *n* = 4; form 2, *n* = 4; and form 3, *n* = 0). The fibrosis stage of the patients with EV was as follows: F0–1, *n* = 0, F2, *n* = 0; F3, *n* = 2; and F4, *n* = 6. The optimum cutoff value (AUROC) for EV form 0 and forms 1–3 was as high as 66.9 (0.883), with a sensitivity and specificity of 87.5% and 86.5%, respectively (Fig. 2d; Table 2).

**Table 2:** Arrival time parametric imaging for assessment of histologic fibrosis stage and predicting the development of paraumbilical veins, splenorenal shunts, esophageal varices, and any of the collateral veins in patients with chronic liver disease

	F2	F3	F4	PV	SRS	EV	CV
Cutoff value (ROR%)	27.5	39.4	60.3	77.9	82.0	66.9	60.3
Sensitivity (%)	77.0	97.6	100.0	100	100	87.5	90.9
Specificity (%)	79.7	90.7	87.7	91.1	94.7	86.5	84.4
AUROC	0.867	0.963	0.965	0.929	0.970	0.883	0.895

*AUROC* area under receiver operating characteristic curve, *F* fibrosis stage, *ROR* ratio of red, *PV* paraumbilical vein, *SRS* splenorenal shunt, *EV* esophageal varices, *CV* any of the collateral veins

### Diagnostic capability for paraumbilical veins based on ROC curves

PV was detected in 2 of the 171 patients (1.2%). The fibrosis stage of the patients with PV was as follows: F0–1, *n* = 0, F2, *n* = 0; F3, *n* = 0; and F4, *n* = 2. The optimum cutoff value (AUROC) for the presence or absence of PV was as high as 77.9 (0.929), with a sensitivity and specificity of 100% and 91.1%, respectively (Fig. 2e; Table 2).

### Diagnostic capability for splenorenal shunts based on ROC curves

Five of the 171 patients (2.9%) were diagnosed with SRS. The fibrosis stage of the patients with SRS was as follows: F0–1, *n* = 0, F2, *n* = 0; F3, *n* = 0; and F4, *n* = 5. The optimum cutoff value (AUROC) for the presence and absence of SRS was as high as 82.0 (0.970), with a sensitivity and specificity of 100% and 94.7%, respectively (Fig. 2f; Table 2).

### Diagnostic capability for any of the collateral veins based on ROC curves

The diagnostic capability for any of the collateral veins (esophageal varices, paraumbilical veins, and splenorenal shunts) was satisfactory with AUROC of 0.895, sensitivity of 90.9%, and specificity of 84.4% at the optimum cutoff value of 60.3% (Fig. 2g; Table 2).

## Discussion

The prevalence of chronic liver disease is increasing worldwide and is an important clinical problem because it is a high-risk factor for the development of PH and hepatocellular carcinoma [13–15]. In the liver, HCV infection advances the disease stage from hepatitis to cirrhosis after repeated necrosis, loss, and fibrosis of liver cells. Unlike the hepatic artery, the hepatic portal vein is susceptible to such changes in liver tissue due to the low-pressure system, and blood pressure increases in line with disease progression. Increasing pressure in the portal vein facilitates the development of collateral vein and serious complications.

In a study by Wakui et al., CEUS was used to compare the contrast dynamics of the liver with those of the kidney supplied by the renal artery and successfully observed changes in blood balance unique to the liver of patients with CHC [6]. The time needed to contrast the liver in hepatitis C patients after contrast enhancement of the kidney was significantly reduced compared with normal individuals as the disease stage progressed from hepatitis to cirrhosis. This finding suggested that blood flow through the portal vein decreases with disease progression toward cirrhosis, and as a compensatory mechanism, the hepatic artery increases the blood flow to achieve hepatic arterial dominance, making hemodynamics in the liver parenchyma similar to that in the kidney. These results indicated that the technique could be an effective noninvasive diagnostic tool for progress of liver fibrosis [6].

PH worsens as liver fibrosis progresses [7] and is a frequent complication of cirrhosis, contributing to the development of collateral veins. The best available methodology for the assessment of PH is measuring the hepatic venous pressure gradient (HVPG). However, HVPG measurement is only possible in highly specialized centers and requires experience, limiting its routine use [8] Therefore, in this study, we investigated whether At-PI predicts the development of collateral veins. Our findings showed that the presence of EV was predicted satisfactorily with AUROC of 0.883, sensitivity of 87.5%, and specificity of 86.5% at the optimum cutoff value of 66.9%. It is common to perform UGI endoscopy for EV. A national survey conducted in Japan revealed that the rate of accidents during UGI endoscopy was 0.057% with a mortality of 0.0002% [16]. Even though the number of accidents is small, to avoid this problem, noninvasive US-based techniques measuring the stiffness of the liver and spleen have been developed to facilitate the diagnosis of EV [17–20]. Takuma et al. investigated the efficacy of acoustic radiation force impulse for diagnosing esophageal varices using spleen stiffness as the indicator in 340 patients with cirrhosis [19] and obtained a cutoff value of 3.18, AUROC of 0.933, sensitivity of 98.5%, and specificity of 60.1%. In a study by Antonio et al., FibroScan was used to examine liver stiffness in 100 hepatitis C patients with cirrhosis and had a cutoff value of 16.4, AUROC of 0.899, sensitivity of 96.2%, and specificity of 59.6% [20]. Kazemi et al. also examined liver stiffness in 175 patients with cirrhosis using FibroScan and reported that the diagnostic capability for EV had a cutoff value of 13.9, AUROC of 0.84, sensitivity of 95.0%, and specificity of 43.0% [17]. Because only one group of patients with cirrhosis was examined in these studies [17–20] and the etiology was not limited to HCV in three of the four studies [17–19], it is difficult to compare these results with those of the present study. Nonetheless, outcome in the present study was as good as that seen in the previous studies.

It is impractical to compare the present findings with previous ones because no previous study used noninvasive US techniques such as elastography to evaluate the diagnostic capability for PV or SRS. Nonetheless, the findings of this study showed that the diagnostic capability for PV and SRS were both especially beneficial. Also, the diagnostic capability for any of the three representative collateral veins was also good. These findings suggest that At-PI is particularly useful for the diagnosis of PV and SRS. It is also clear that collateral venous circulation develops when ROR (arterialization ratio) is ≥ 60.3%. This also suggests that the development of collateral veins can be predicted from ROR calculated using At-PI.

We think these satisfactory data are attributable to the special features of the present technique. Our technique is designed to visualize differences in the arrival time of a contrast agent to the liver and kidney, to identify the ever-changing hepatic blood flow (i.e., the hepatic blood balance between the hepatic artery and portal vein due to disease progression). It is possible that blood flow visualization in the entire right hepatic lobe led to the direct assessment of collateral venous circulation, with good outcomes.

There were, however, several limitations to this study. The following diseases and conditions may affect the results of our method: heart disease associated with possible alteration of the arrival time of the contrast agent to the liver, renal disorders associated with possible alterations of US signal kinetics in the kidney, habitual heavy drinking associated with possible changes in hemodynamics, and portal vein embolism associated with possible disturbance of the balance between arterial and portal blood flow. Thus, patients with these diseases or conditions cannot be examined by this method. Furthermore, patients in whom the right hepatic lobe cannot be visualized by sonography, including those with narrow intercostal spaces, and those who experience difficulty holding their breath for 15–20 s, must be excluded. Because perflubutane contains an egg derivative, egg allergy is a contraindication.

In Japan, medical expenses associated with CEUS are 1.6 times higher than those for upper gastrointestinal endoscopy. However, the present technique is not only noninvasive and repeatable, but also useful for obtaining information that can assist in the diagnosis of, for example, liver fibrosis.

In conclusion, this study investigated the diagnostic capability of At-PI and demonstrated that collateral veins develop in patients with CHC at the ROR of ≥ 60.3%, suggesting that the evaluation of hepatic arterialization by At-PI is useful in predicting the development of collateral veins.
